# Career, Autonomy, Rights and Equity among Doctors: Brazilian Medical Association survey

**DOI:** 10.1590/1806-9282.20261603

**Published:** 2026-11-13

**Authors:** Luciana Rodrigues Silva, Maria Rita de Souza Mesquita, Claudia Navarro Carvalho Duarte Lemus, Alex Jones Flores Cassenote, Mateus de Souza Ribeiro, José Eduardo Lutaif Dolci, Cesar Eduardo Fernandes

**Affiliations:** 1 Brazilian Medical Association – São Paulo (SP), Brazil.; 2 Universidade Federal da Bahia – Salvador (BA), Brazil.; 3 Universidade Federal de São Paulo – São Paulo (SP), Brazil.; 4 Universidade de São Paulo, Institute of Advanced Studies – São Paulo (SP), Brazil.; 5 Universidade Brasil – Fernandópolis (SP), Brazil.; 6 Santa Marcelina College, School of Medicine – São Paulo (SP), Brazil.; 7 Santa Casa de São Paulo School of Medical Sciences – São Paulo (SP), Brazil.; 8 ABC Medical School – Santo André (SP), Brazil.

**Keywords:** Physicians, Surveys and questionnaires, Gender equity, Health workforce, Data linkage, Brazil

## Abstract

**OBJECTIVE:**

This study aimed to describe the design and linkage procedures of the Career, Autonomy, Rights and Equity among Doctors Brazil survey coordinated by the Brazilian Medical Association, and to assess the composition and geographic coverage of its analytic sample against national benchmarks.

**METHODS:**

This national cross-sectional online survey was conducted from January 29 to September 10, 2026. The invitation frame came from Demografia Médica no Brasil, a census-based investigation of the physician workforce conducted since 2010. Four nonoverlapping random samples totaling 110,000 physicians were invited; participation was voluntary. Survey records were audited, deduplicated, linked to professional sources through a restricted key, compared with 2024 national benchmarks, and calibrated for sex, region, and age group.

**RESULTS:**

Of 4,437 submissions, 4,002 physicians formed the analytic sample. Women accounted for 51.6%, mean age was 48.3 years, and 70.9% had at least one recognized specialty, compared with 49.3%, 44.8 years, and 59.1% in the observed 2024 national benchmark. Calibration weights ranged from 0.396 to 3.134; the Kish weight-based design effect was 1.259, and the effective sample size was 3,180.

**CONCLUSION:**

Career, Autonomy, Rights and Equity among Doctors Brazil documents a linked physician sample and quantifies differences from national benchmarks. The low matched participation fraction and voluntary response create substantial potential for selection bias. Random invitations, linkage, and calibration do not establish population representativeness or remove unmeasured nonresponse bias. The findings primarily characterize the analytic sample and inform the interpretation of subsequent thematic studies.

## INTRODUCTION

Women are projected to account for more than half of Brazil's physicians in 2026^1^. This numerical majority does not ensure equity in pay, working conditions, specialty choice, leadership, or career progression. Mainardi et al. investigated remuneration; Amaral et al. and Suzuki et al. studied workplace discrimination; Motter et al. examined surgical leadership; and Marhoffer et al. reviewed gender in academic promotion^2-6^. Together, these studies do not provide a shared participant base for comparing several career dimensions under one recruitment and linkage design.

These limitations matter for a voluntary online survey. Random invitation does not make respondents a probability sample; response, linkage, and missingness may change each analytic subset. External census comparisons and calibration clarify the reach and limits of inference but cannot correct unmeasured nonresponse. Reporting these steps supports comparable thematic analyses of gender, work, and income^7-11^.

The methodological gap is how to define and evaluate a common analytic universe for focused studies with consistent denominators, linked professional information, and explicit limits to generalization. Career, Autonomy, Rights and Equity among Doctors (CARE-DOC) Brazil links measures of work, family, autonomy, discrimination, leadership, remuneration, and future expectations to professional information. This article describes the instrument, recruitment, processing, and linked sample, and empirically assesses deviations from national benchmarks, geographic coverage, and calibration-weight dispersion. Its contribution is descriptive and methodological; the potential for future thematic research is a secondary application.

## METHODS

### Study design and instrument

CARE-DOC Brazil was a national cross-sectional online survey coordinated by the Brazilian Medical Association from January 29 to September 10, 2026. The Portuguese questionnaire was developed through literature review, institutional discussion, and version review, drawing on Brazilian physician studies^1,2^. It combined single- and multiple-choice, numeric, and five-point agreement items across work, family, labor rights, discrimination, leadership, gender attitudes, and future plans. For up to seven regular medical work relationships, the repeatable module recorded care setting, financing (public Brazilian Unified Health System, insurance, or direct payment), contract, leadership, activity, weekly hours, and monthly net-income band. Contracts included employment under Brazilian labor law (Consolidation of Labor Laws), statutory public service, contracting through a legal entity (pessoa jurídica, PJ), independent practice, and residency or fellowship stipends. Twenty selected Ambivalent Sexism Inventory statements and four modified work–family conflict statements used five-point agreement responses^12,13^. Their wording and response formats were not assumed equivalent to the source instruments. Formal cognitive testing and prefield psychometric validation were not conducted; any composite score requires separate evaluation.

Reporting followed Consensus-Based Checklist for Reporting of Survey Studies, Checklist for Reporting Results of Internet E-Surveys, and Strengthening the Reporting of Observational Studies in Epidemiology guidance^7,8,14^. Figure 1 presents the audited sample flow.

**Figure 1:**
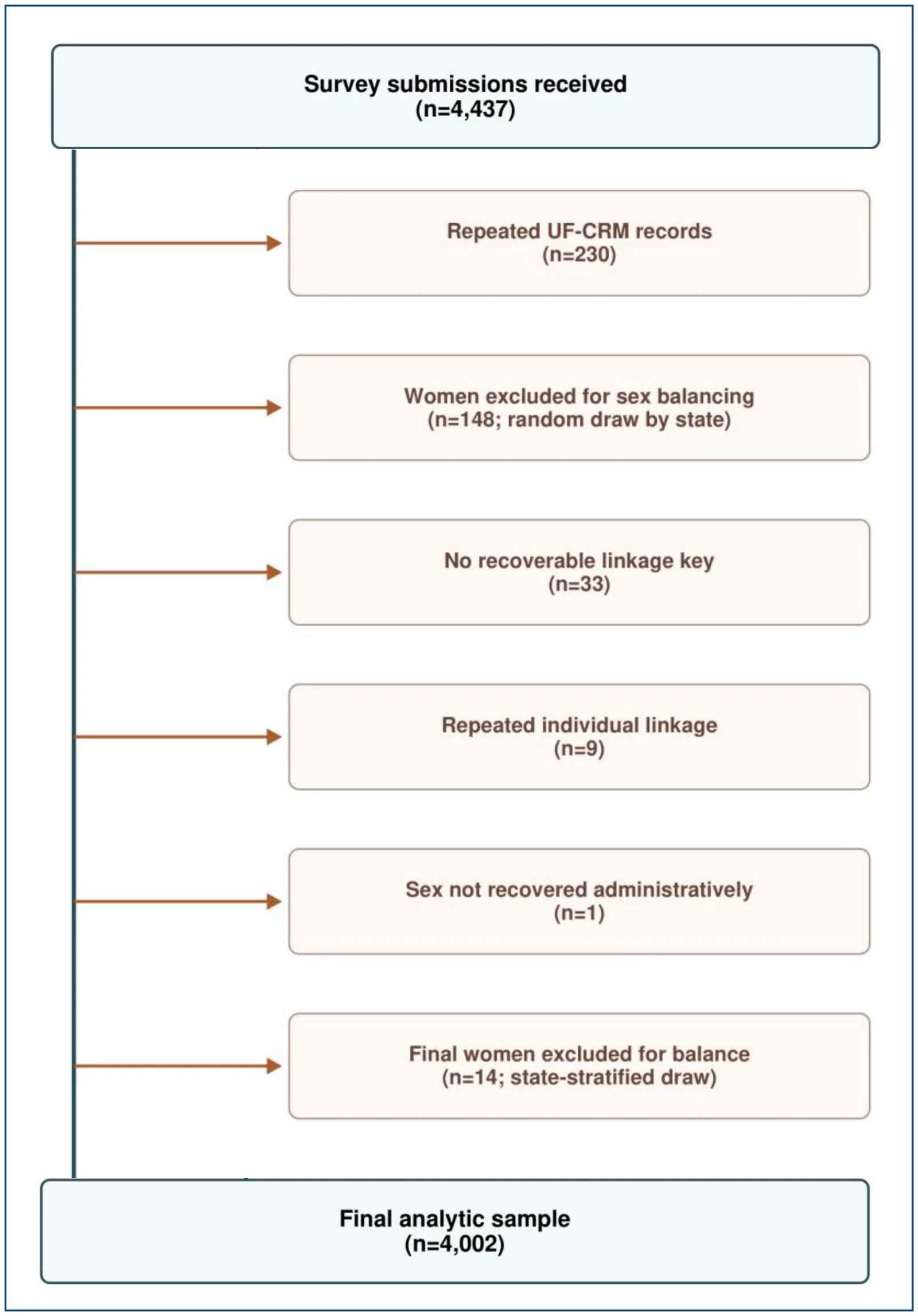
Formation of the final analytic sample. The flow records all exclusions from 4,437 submissions to 4,002 participants. Direct identifiers were excluded from analytic outputs.

### Recruitment, linkage, and privacy

The invitation frame came from Demografia Médica no Brasil, a nationwide census-based investigation conducted since 2010^1^. Four nonoverlapping random invitation waves of 10,000, 20,000, 30,000, and 50,000 physicians totaled 110,000; participation was voluntary. The Brazilian Medical Association coordinated mobilization, not the frame. Among 4,437 submissions, we kept the more complete of repeated federative unit-Regional Council of Medicine registration pair records (most recent if tied), removing 230. Seven initially had no male/female sex classification. After a provisional geographic balancing step, 148 women were randomly excluded proportionally across adjusted-state strata from the remaining 4,200 (seed 20260919) to reduce the female excess, leaving 4,052. We then excluded 33 without a recoverable linkage key and nine repeated individual linkages. Six of the seven sex-unclassified records were recovered from administrative data; one remained excluded. Finally, 14 women (12 in São Paulo, one each in Minas Gerais and Rio Grande do Sul) were randomly excluded within provisional state strata (seed 20260921), yielding 4,002. Of these, 3,939 matched the invitation frame and 63 did not. Before analysis, we compared excluded and retained self-reported women on raw questionnaire characteristics.

Deduplication and deterministic linkage used a restricted key available only to the data-management team. Linked sources supplied or verified age, graduation, school sector, specialty and area-of-practice records, residency, and specialty-board certification. Registry data corrected the analysis sex classification for 45 records; original self-reports were retained separately. Direct identifiers were excluded from analysis. Absence from a title registry was coded as no recorded specialty or practice area.

### Study size and participation

The analytic sample included consenting physicians with a unique recoverable linkage key and sex; matching the invitation list was not required. As a descriptive precision benchmark, the finite-population formula with 597,428 physicians in 2024, 50% expected prevalence, 95% confidence, and a two-percentage-point margin under simple random sampling yielded 2,392 physicians^15^. This benchmark does not establish achieved precision, population representativeness, or power for thematic outcomes. Of 110,000 selected invitees, 3,939 appeared in the analytic sample (3.6%). This matched participation fraction is not an American Association for Public Opinion Research response rate: invitation delivery and eligibility among nonrespondents were unverified^16^. The final analytic sample also included 63 physicians without a match to the invitation frame. Thus, dividing all 4,002 participants by 110,000 invitees (3.64%) does not estimate participation among sampled invitees. Neither fraction establishes that respondents resemble nonrespondents.

### Population comparisons and statistical analysis

The principal benchmark was Demografia Médica no Brasil 2025, which describes physicians and active registrations in 2024^1^. Sex, mean age, and specialty were compared with individual-physician estimates. Regional comparisons used participants’ original reported state and active registrations, which may include more than one registration per physician. The provisional balancing file reassigned 103 retained participants to other state strata; final geography and weights use their original state. School sector and time since graduation came from the linked administrative extract dated October 10, 2024. Age and time since graduation were standardized to December 31, 2024. For the municipality map, a restricted key linked respondents to the 2024 physician registry. We selected an exact state-and-registration match or an unambiguous municipality within the original state, plotted aggregate counts within Brazilian Institute of Geography and Statistics (IBGE) municipal boundaries, and omitted ambiguous addresses. Registry correspondence address may differ from residence or practice location.

We calculated Wilson 95%CIs for proportions, a t-based interval for mean age, standardized differences, and state observed-to-expected ratios (original-state survey share divided by the national active-registration share). Pearson goodness-of-fit tests assessed sex, five regions, and age groups (<35, 35–44, 45–54, 55–64, and ≥65 years). Intervals and p-values assume simple random sampling and do not account for voluntary response or post-collection selection. Calibration weights were estimated by iterative proportional fitting to 2024 sex totals, age groups among physicians with known age, and regional active-registration totals, using the original state^9^. Seven missing ages were assigned the sex-specific sample median only for calibration; descriptive age used 3,995 observed values. Weights were normalized to mean one. Descriptive estimates used available cases and variable-specific denominators. We reported weight range, coefficient of variation (CV), Kish weight-based design effect (DEFF)=1+CV^2^, and effective n=n/DEFF^17^. This DEFF describes weight dispersion, not the full sampling variance or nonresponse bias. The effective sample size is therefore a weight-based information benchmark, not evidence of unbiased population estimates. Calibration aligns the selected observed margins but cannot establish exchangeability between respondents and nonrespondents or correct selection related to unmeasured characteristics. Analyses used R version 4.6.1 (R Foundation for Statistical Computing, Vienna, Austria).

### Ethics

The Research Ethics Committee of the University of São Paulo School of Medicine approved the study (Certificate of Presentation for Ethical Consideration 71626323.8.0000.0068). Participation was voluntary and electronic informed consent was obtained before questionnaire completion.

## RESULTS

### Analytic sample and external comparison

The final sample comprised 4,002 physicians (90.2% of submissions), including 3,939 matched to the invitation frame (3.58% of 110,000 invitees) and 63 without a frame match. Mean questionnaire completeness was 99.0% among included participants; it does not indicate high participation among invitees. Women represented 51.6% (95%CI 50.1–53.2). Mean age was 48.3 years (standard deviation [SD] 14.3; 95%CI 47.9–48.7), 3.5 years above the national mean. At least one recognized specialty was recorded for 70.9% of participants versus 59.1% nationally. The sample also contained more public-school graduates and longer-term graduates (Table 1).

**Table 1:** Participant profile and comparison with national physician benchmarks.

Characteristic	Survey n/N	Estimate	95%CI	DMB 2024	Difference
Women	2,066/4,002	51.6%	50.1–53.2	49.3%	+2.3 pp
Age, mean (SD), years	3,995/4,002	48.3 (14.3)	47.9–48.7	44.8 (15.7)	+3.5 years
Black or brown	653/3,985	16.4%	15.3–17.6	–	–
North	130/4,002	3.2%	2.7–3.8	4.8%	-1.6 pp
Northeast	568/4,002	14.2%	13.1–15.3	19.3%	-5.1 pp
Southeast	2,209/4,002	55.2%	53.7–56.7	51.1%	+4.1 pp
South	788/4,002	19.7%	18.5–21.0	15.8%	+3.9 pp
Central-West	307/4,002	7.7%	6.9–8.5	9.0%	-1.3 pp
Public medical school	2,244/3,887	57.7%	56.2–59.3	53.7%	+4.1 pp
No recognized specialty	1,163/4,002	29.1%	27.7–30.5	40.9%	-11.8 pp
One recognized specialty	1,858/4,002	46.4%	44.9–48.0	40.9%	+5.5 pp
Two recognized specialties	821/4,002	20.5%	19.3–21.8	16.0%	+4.5 pp
Three or more specialties	160/4,002	4.0%	3.4–4.7	2.2%	+1.8 pp

CI: confidence interval; DMB: Demografia Médica no Brasil; pp: percentage points; SD: standard deviation. Regional benchmarks use active registrations. A dash indicates no comparator.

### Geographic coverage and calibration

All 27 federative units were represented. The North and Northeast were underrepresented by 1.6 and 5.1 percentage points, whereas the Southeast and South were overrepresented by 4.1 and 3.9 points. Goodness-of-fit tests identified differences for sex (chi-square[1]=8.65; p=0.003), original-state region (chi-square[4]=126.84; p<0.001), and age group (chi-square[4]=799.04; p<0.001). Raking converged in 25 iterations. Weights had mean 1.000, CV 0.508, range 0.396–3.134, and 1st–99th percentiles 0.396–2.493. The Kish weight-based DEFF was 1.259 and effective sample size was 3,180. Among 162 excluded versus 2,073 retained self-reported women, 38.3% versus 36.2% reported at least three jobs, and 20.4% versus 17.3% reported Black/Brown race or color.

For 3,928 of 4,002 participants (98.2%), the correspondence-address municipality could be linked unambiguously within the original survey state; they represented 648 municipalities. The remaining 74 were not mapped (50 registry records only in another state, 20 without a municipality code, and four without a registry match). Panel B compares each original state's share of the survey with its share of active physician registrations in the national benchmark (Figure 2).

**Figure 2:**
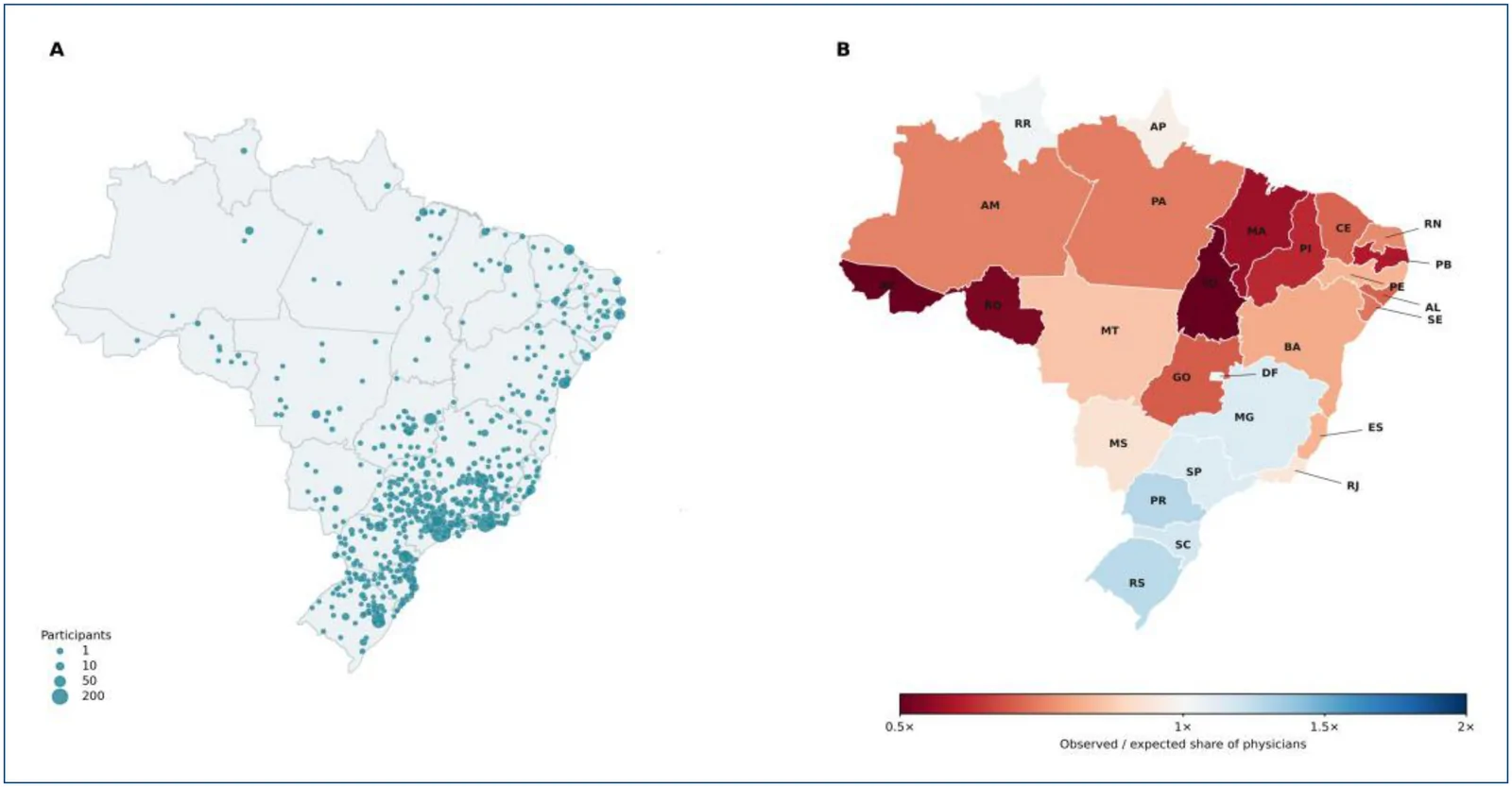
Geographic distribution of linked participants and state-level relative participation. (A) Aggregate number of linked participants by municipality of correspondence address in the October 10, 2024 physician registry, plotted within 2024 Brazilian Institute of Geography and Statistics municipal boundaries; 3,928 participants in 648 municipalities. The address does not necessarily indicate residence or practice location. (B) Observed-to-expected ratios using participants’ original state; values above 1 indicate a larger survey share than the national share. The color scale is capped at 0.5 and 2.0. State boundaries: IBGE 2024. The Demografia Médica no Brasil 2025 benchmark reports active registrations in 2024.

## DISCUSSION

CARE-DOC Brazil documents the construction and composition of a linked physician sample and demonstrates measurable deviations from national benchmarks. Respondents were older and more frequently specialized, and their regional distribution differed from the benchmark. The calibration diagnostics quantify the dispersion of weights used to align selected margins. These are descriptive methodological findings about the observed sample. The linked database may support thematic studies, but that future utility is not itself evidence of substantive findings on working conditions, gender equity, or health-service outcomes in the present article.

The principal limitation is the very low matched participation fraction: only 3,939 of 110,000 invited physicians entered the analytic sample. Random selection strengthens the invitation framework but does not ensure a representative final sample when participation is voluntary. Respondents may differ systematically from nonrespondents in professional engagement, available time, experiences of discrimination, work–family conflict, or interest in the survey topic. These are plausible selection mechanisms, not differences demonstrated by our data. Their direction and magnitude cannot be determined from the available respondent data, and substantial nonresponse selection bias remains possible^10^. Mobilization through specialty groups may also have contributed to the older and more specialized respondent profile, but this cannot be quantified.

Calibration aligned sex, age-group, and regional margins with the chosen 2024 benchmarks^9^. This improves comparability on those observed dimensions but cannot eliminate bias associated with unmeasured determinants of participation or outcomes. Regional targets based on active registrations also differ from individual-physician totals, and the benchmarks precede the survey by two years. The Kish weight-based DEFF of 1.259 describes weight dispersion and does not measure total uncertainty or residual selection bias. Thematic studies should present unweighted estimates alongside calibrated sensitivity analyses and report weight dispersion and effective sample size. Agreement between weighted and unweighted estimates would show robustness to the specified calibration, not proof of population validity. Linkage reduced respondent burden and supported classification of career attributes, but it can also generate selection and classification error^11^. Analyses should retain match indicators, document registry rules, and compare linked and unlinked records when relevant. Adapted multi-item constructs require reliability and dimensional assessment before composite scores are compared; the survey as a whole is not a diagnostic or psychometric scale.

Additional limitations include post-collection exclusion of 162 women to balance sex and state, 63 participants unmatched to the invitation frame, differences between 2024 registry data and 2026 responses, self-report error, and no formal prefield cognitive interviewing. Post-collection exclusions may further alter the composition of the sample and thematic estimates. High questionnaire completeness and audited linkage improve data quality among included participants but do not resolve selection into that sample. Representation of all states establishes geographic presence, not representative coverage of physicians within each state. The predominantly descriptive design limits the scientific contribution to documenting sample construction and assessing its observed composition and inferential constraints. It does not estimate causal effects or demonstrate future consequences for physicians or health services. Population prevalence estimates and associations derived from this resource require explicit qualification because calibration cannot justify unrestricted national inference.

## CONCLUSION

CARE-DOC Brazil provides a documented linked analytic sample of 4,002 physicians and an empirical assessment of its composition, geographic presence, and calibration diagnostics. The low matched participation fraction, voluntary response, and post-collection selection limit generalizability. Random invitation and administrative linkage do not overcome these limitations, and calibration cannot remove unmeasured nonresponse bias. Subsequent thematic studies can use this resource to examine patterns among participants, with calibrated sensitivity analyses and explicit limits on population interpretation.

## Data Availability

De-identified data may be made available from the corresponding author upon reasonable request, subject to applicable ethical and confidentiality restrictions and institutional authorization. Identifiable data will not be shared.
